# Supplementary material for: Oxytocin modulates human chemosensory decoding of sex in a dose-dependent manner
Source: eLife. 2021 Jan 13;10:e59376. doi: 10.7554/eLife.59376 (PMC7806258; doi:10.7554/eLife.59376)
Supplement: Transparent reporting form [file elife-59376-transrepform.docx]

***eLife’s* transparent reporting form**

We encourage authors to provide detailed information *within their submission* to facilitate the interpretation and replication of experiments. Authors can upload supporting documentation to indicate the use of appropriate reporting guidelines for health-related research (see [EQUATOR Network](http://www.equator-network.org/%20)), life science research (see the [BioSharing Information Resource](https://biosharing.org/)), or the [ARRIVE guidelines](http://www.plosbiology.org/article/info:doi/10.1371/journal.pbio.1000412) for reporting work involving animal research. Where applicable, authors should refer to any relevant reporting standards documents in this form.

If you have any questions, please consult our Journal Policies and/or contact us: [editorial@elifesciences.org](mailto:editorial@elifesciences.org).

**Sample-size estimation**

- You should state whether an appropriate sample size was computed when the study was being designed
- You should state the statistical method of sample size computation and any required assumptions
- If no explicit power analysis was used, you should describe how you decided what sample (replicate) size (number) to use

Please outline where this information can be found within the submission (e.g., sections or figure legends), or explain why this information doesn’t apply to your submission:

Sample sizes (n = 24 in each subgroup) were determined by G*Power to be adequate to detect a moderate effect of androstadienone or estratetraenol (d ≈ 0.6), at 80% power. The effect size was estimated based on an earlier study that employed almost identical stimuli and psychophysical testing procedures to those in the current study (Zhou et al., 2014). These are mentioned in the Participants section under Materials and Methods.

**Replicates**

- You should report how often each experiment was performed
- You should include a definition of biological versus technical replication
- The data obtained should be provided and sufficient information should be provided to indicate the number of independent biological and/or technical replicates
- If you encountered any outliers, you should describe how these were handled
- Criteria for exclusion/inclusion of data should be clearly stated
- High-throughput sequence data should be uploaded before submission, with a private link for reviewers provided (these are available from both GEO and ArrayExpress)

Please outline where this information can be found within the submission (e.g., sections or figure legends), or explain why this information doesn’t apply to your submission:

Each experiment was performed once. Experiments 1 and 2 suggested that the decoding of chemosensory sexual information involved oxytocin and not vasopressin. This finding was consistently replicated in Experiments 3-5. In addition, Experiment 3 showed a nonmonotonic effect of oxytocin on chemosensory decoding of sex. This was fully replicated in Experiment 4. See Table 1 for summary.

**Statistical reporting**

- Statistical analysis methods should be described and justified
- Raw data should be presented in figures whenever informative to do so (typically when N per group is less than 10)
- For each experiment, you should identify the statistical tests used, exact values of N, definitions of center, methods of multiple test correction, and dispersion and precision measures (e.g., mean, median, SD, SEM, confidence intervals; and, for the major substantive results, a measure of effect size (e.g., Pearson's r, Cohen's d)
- Report exact p-values wherever possible alongside the summary statistics and 95% confidence intervals. These should be reported for all key questions and not only when the p-value is less than 0.05.

Please outline where this information can be found within the submission (e.g., sections or figure legends), or explain why this information doesn’t apply to your submission:

Data analyses are described in Materials and Methods – Statistical analysis. Detailed statistical results are reported in the Results section (Figures 2-5 and their figure supplements). Experiment 4 is a replication of Experiment 3. Statistical results for Experiment 4 are included in the legend for Figure 4-figure supplement 1.

(For large datasets, or papers with a very large number of statistical tests, you may upload a single table file with tests, Ns, etc., with reference to sections in the manuscript.)

**Group allocation**

- Indicate how samples were allocated into experimental groups (in the case of clinical studies, please specify allocation to treatment method); if randomization was used, please also state if restricted randomization was applied
- Indicate if masking was used during group allocation, data collection and/or data analysis

Please outline where this information can be found within the submission (e.g., sections or figure legends), or explain why this information doesn’t apply to your submission:

In Experiments 1 and 2, participants were randomly assigned to 3 groups of 24 each to receive different intranasal drug treatments in a double-blind procedure. Experiments 3-5 employed a within-subjects design and did not involve group allocation. These are stated in the Results section and illustrated in Figure 1. The olfactory stimuli and drug solutions in each experiment were coded by an individual not involved in the study. This is stated in the Olfactory stimuli and Drug application sections under Materials and Methods.

**Additional data files (“source data”)**

- We encourage you to upload relevant additional data files, such as numerical data that are represented as a graph in a figure, or as a summary table
- Where provided, these should be in the most useful format, and they can be uploaded as “Source data” files linked to a main figure or table
- Include model definition files including the full list of parameters used
- Include code used for data analysis (e.g., R, MatLab)
- Avoid stating that data files are “available upon request”

Please indicate the figures or tables for which source data files have been provided:

All group-level statistics are plotted in Figures 2-5. Source data files have been provided for Figures 2, 3, and 4. Please note that Figure 5 summarizes the results of Experiments 3-5 and is based on the same source data files as Figure 4.
